# CRISPR/Cas9-mediated mutation of *FERULATE 5-HYDROXYLASE* enhances adsorption capacity of lignocellulose-based porous carbon from paper mulberry

**DOI:** 10.1093/hr/uhae124

**Published:** 2024-04-23

**Authors:** Yue Xu, Yingping Cao, Wanju Zhang, Wen Kong, Rui Li, Yuchen Liu, Yu Wang, Zhenying Wu, Xiaochun Qin, Feng He, Chunxiang Fu

**Affiliations:** School of Chemistry and Chemical Engineering, University of Jinan, Jinan 250022, China; Shandong Provincial Key Laboratory of Energy Genetics, Qingdao Institute of Bioenergy and Bioprocess Technology, Chinese Academy of Sciences, Qingdao 266101, China; Shandong Energy Institute, Qingdao 266101, China; Qingdao New Energy Shandong Laboratory, Qingdao 266101, China; Shandong Provincial Key Laboratory of Energy Genetics, Qingdao Institute of Bioenergy and Bioprocess Technology, Chinese Academy of Sciences, Qingdao 266101, China; Shandong Energy Institute, Qingdao 266101, China; Qingdao New Energy Shandong Laboratory, Qingdao 266101, China; LiShizhen College of Traditional Chinese Medicine, Huanggang Normal University, Huanggang 438000, China; LiShizhen College of Traditional Chinese Medicine, Huanggang Normal University, Huanggang 438000, China; Shandong Provincial Key Laboratory of Energy Genetics, Qingdao Institute of Bioenergy and Bioprocess Technology, Chinese Academy of Sciences, Qingdao 266101, China; Shandong Energy Institute, Qingdao 266101, China; Qingdao New Energy Shandong Laboratory, Qingdao 266101, China; Shandong Provincial Key Laboratory of Energy Genetics, Qingdao Institute of Bioenergy and Bioprocess Technology, Chinese Academy of Sciences, Qingdao 266101, China; Shandong Energy Institute, Qingdao 266101, China; Qingdao New Energy Shandong Laboratory, Qingdao 266101, China; Shandong Provincial Key Laboratory of Energy Genetics, Qingdao Institute of Bioenergy and Bioprocess Technology, Chinese Academy of Sciences, Qingdao 266101, China; Shandong Energy Institute, Qingdao 266101, China; Qingdao New Energy Shandong Laboratory, Qingdao 266101, China; Shandong Provincial Key Laboratory of Energy Genetics, Qingdao Institute of Bioenergy and Bioprocess Technology, Chinese Academy of Sciences, Qingdao 266101, China; Shandong Energy Institute, Qingdao 266101, China; Qingdao New Energy Shandong Laboratory, Qingdao 266101, China; School of Chemistry and Chemical Engineering, University of Jinan, Jinan 250022, China; Shandong Provincial Key Laboratory of Energy Genetics, Qingdao Institute of Bioenergy and Bioprocess Technology, Chinese Academy of Sciences, Qingdao 266101, China; Shandong Energy Institute, Qingdao 266101, China; Qingdao New Energy Shandong Laboratory, Qingdao 266101, China; Shandong Provincial Key Laboratory of Energy Genetics, Qingdao Institute of Bioenergy and Bioprocess Technology, Chinese Academy of Sciences, Qingdao 266101, China; Shandong Energy Institute, Qingdao 266101, China; Qingdao New Energy Shandong Laboratory, Qingdao 266101, China; CAS Key Laboratory of Tibetan Medicine Research, Northwest Institute of Plateau Biology, Xining 810008, China

Dear Editor,

Paper mulberry (*Broussonetia papyrifera*, 2*n* = 2*x* = 26) is a fast-growing ornamental tree known for its significant ecological, economic, forage, and medicinal values, and is cultivated or naturalized across tropical and subtropical regions worldwide [1]. Pruning is one of the most important cultural practices for maintaining paper mulberry plants. This horticultural waste provides large amounts of lignocellulosic biomass, which can be utilized for producing high value-added carbon materials [2]. The lignin polymers of paper mulberry are rich in syringyl (S) units followed by guaiacyl (G) units and traces of *p*-hydroxyphenyl (H) units, bonded through various C-O (e.g. β-*O*-4, α-*O*-4, and 4-*O*-5) and C-C (e.g. β-β and β-5,5-5) linkages [3]. In recent years, the production of porous carbon materials from lignocellulosic biomass has gained attention due to its cost-effectiveness, high value, sustainability, and renewable nature [4]. However, the effect of lignin composition on the porous structure of biocarbon materials remains largely unknown. Encouragingly, recent studies indicate that genetic manipulation of lignin is an efficient strategy in advancing lignin valorization technology in woody plants [5]. Thus, we produced paper mulberry materials with distinct lignin compositions through the overexpression and CRISPR/Cas9-mediated gene editing of the crucial gene responsible for S lignin biosynthesis. The impact of G and S units on the porosity and adsorption capacity of paper mulberry-derived porous carbons was comprehensively evaluated.

**Figure 1:**
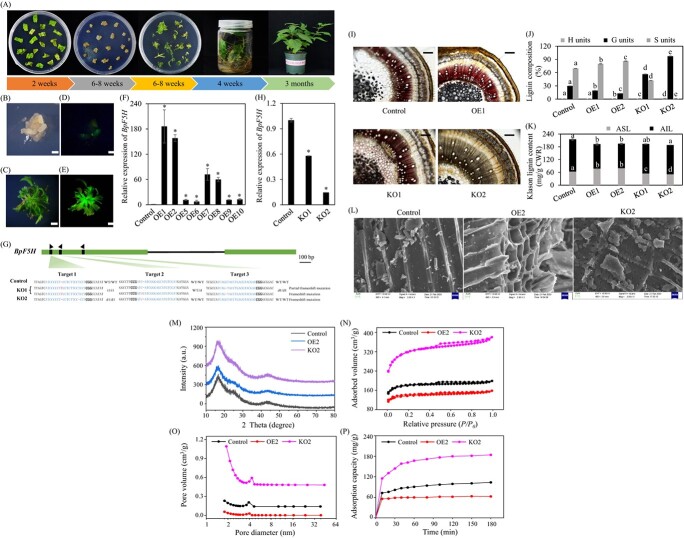
Genetic modification of *BpF5H* for enhanced quality as a porous carbon resource for methyl orange absorption. **A** Schematic representation of the transformation process to generate *BpF5H* transgenic lines. **B**–**E** Microscopic investigation and GFP signal detection of transformed calli and regenerated shoots. Scale bar = 1.5 mm. **F** Expression level of *BpF5H* in the *BpF5H* overexpression (OE) lines. **G** Gene structure of *BpF5H* and genotyping of biallelic *BpF5H* mutants. PAM sequences are in bold. I1 and d1 represent 1-bp insertion and 1-bp deletion in the target sites, respectively. **H** Transcript abundance of *BpF5H* in the *BpF5H* knockout (KO) lines. **I** Mäule staining of stem cross-sections from the control, OE, and KO plants. Scale bar = 500 μm. **J**, **K** Lignin content and composition. **L** Scanning electron microscope images of porous carbon. **M** X-ray diffraction patterns. **N** N_2_ adsorption–desorption isotherm. **O** Pore size distribution of biochar. **P** Adsorption performance of porous carbon from the control, OE, and KO plants. Values are means ± standard error (*n* = 3). One-way ANOVA with Dunnett’s multiple range test was used in **F** and **H**; *P < 0.05, **P < 0.01. Letters above the error bars in **J** and **K** indicate significant differences determined by one-way ANOVA (*P* < 0.05, Duncan’s multiple range test). Control plants were transformed with the pEarlyGate100-eGFP empty vector. CWR, cell wall residue; ASL, acid-soluble lignin; AIL, acid-insoluble lignin.

The *ferulate-5-hydroxylase* (*F5H*) gene encodes a crucial enzyme in the conversion of G to S monolignol [6]. To modulate the biosynthesis of S units in lignin, the *F5H* gene in paper mulberry was identified and cloned (Supplementary Data Fig. S1), and a total of 10 independent transgenic paper mulberry lines overexpressing *BpF5H* (BpF5H_OE) were generated using a highly efficient *Agrobacterium*-mediated transgenic system (Fig. 1A–E; Supplementary Data Fig. S2). Quantitative real-time PCR (qRT–PCR) analysis revealed a significant 7- to 186-fold increase of BpF5H expression level in the BpF5H_OE transgenic lines compared with the control plants (Fig. 1F; Supplementary Data Table S1). Additionally, a gene editing system was established in paper mulberry. Among 19 transgenic-positive lines, 15 lines were mutated at the target sites of *BpF5H* (Supplementary Data Table S2). The editing efficiency among the different target sites ranged from 22.0% for target site 2 to 62.0 and 68.0% for target sites 3 and 1, respectively (Supplementary Data Table S2). Sequencing data revealed the editing types of representative homozygous/biallelic mutation lines KO1 and KO2, with no detection of the wild-type allele at the target site of *BpF5H* (Fig. 1G). Interestingly, the biallelic mutation line BpF5H_KO1 exhibited a full frame shift allele (1-bp insertion at both sgRNA1 and sgRNA2) and a partial frame shift allele (1-bp insertion at sgRNA1 and 1-bp deletion at sgRNA3) (Fig. 1G; Supplementary Data Fig. S3; Supplementary Data Table S2). Furthermore, the qRT–PCR analysis demonstrated a modest reduction (42.2%) of *BpF5H* expression level in BpF5H_KO1*,* while a sharper reduction in transcript abundance of *BpF5H* was observed in BpF5H_KO2 (Fig. 1H).

Two transgenic lines (BpF5H_OE1 and BpF5H_OE2) with 186- and 158-fold higher *BpF5H* expression levels and two biallelic mutation lines (BpF5H_KO1 and BpF5H_KO2) were selected for further investigation. Cross-sections of the internodes from control, BpF5H_OE, and BpF5H_KO plants were stained with Mäule reagent. The intensity of red coloration is a good indicator of the amount of S units. Maüle staining showed a change from brown in the control internode to burgundy red around vascular bundles in the BpF5H_OE1 internode, indicating an increase in S units. In contrast, the brownish coloration became lighter in BpF5H_KO1 and had almost disappeared in BpF5H_KO2, revealing a substantial reduction in S units (Fig. 1I). Lignin composition analysis further unveiled a 15–24% increase in S units and a 35–57% decrease in G units in BpF5H_OE lines compared with control plants (Fig. 1J). No S units were detected in BpF5H_KO2, characterized as a complete loss of function in *BpF5H*. In contrast, BpF5H_KO1 exhibited a 39% reduction in S units, suggesting the *BpF5H* gene with a partial frame shift still retains functionality in the biosynthesis of S monolignol (Fig. 1J). Due to the *BpF5H* mutation, BpF5H_KO1 and BpF5H_KO2 produced ~1.9-fold and 3.3-fold more G units than control plants, respectively (Fig. 1J). Klason lignin content analysis demonstrated reduced total lignin contents in both BpF5H_OE and BpF5H_KO plants compared with control plants, consistent with changes in acid-insoluble lignin contents (Fig. 1K). Additionally, acid-soluble lignin contents were increased in the BpF5H_OE lines but decreased in the BpF5H_KO lines (Fig. 1K). These results align with previous observations that S lignin-enriched wood materials dissolve and depolymerize rapidly in 72% sulfuric acid [7].

To investigate the influence of S units on the performance of lignocellulosic biomass-based pyrolysis materials, porous carbon samples were prepared from the control, BpF5H_OE, and BpF5H_KO plants through carbonization at 700°C for 2 h. Scanning electron microscopy analysis revealed a significantly increased number of pits in the carbonized cell walls of the BpF5H_KO2 compared with the control and BpF5H_OE2 samples (Fig. 1L). Additionally, the X-ray diffraction pattern assay showed an increased broad diffraction peak around 17° in BpF5H_KO2, indicating a higher degree of amorphous structure (Fig. 1M). Generally, all porous carbons derived from paper mulberry exhibited combined characteristics of type IV isotherms with a hysteresis loop (Fig. 1N). Notably, BpF5H_KO2-derived porous carbon displayed the highest N_2_ adsorption capacity, with a significant increase in adsorbed volume at a relative pressure <0.05, suggesting the presence of a greater number of micropores (Fig. 1O; Supplementary Data Table S3). The pore size distribution assay demonstrated a substantial increase in the volume of micropores in BpF5H_KO2-derived porous carbon, with the surface area reaching 1000.87 m^2^/g, the highest among all samples (Fig. 1O). In comparison, the BpF5H_OE2-derived porous carbon exhibited fewer micropores and a smaller adsorbed volume (Fig. 1N and O). When exposed to methyl orange (MO) solution, the BpF5H_KO2-derived porous carbon exhibited an absorption capacity of nearly 180 mg/g after 3 h, which was 2-fold higher than that of the control sample (Fig. 1P). As anticipated, a lower absorption capacity (62.6 mg/g after 3 h) was observed in the BpF5H_OE2-derived porous carbon. In our work, the effect of *F5H* mutation in paper mulberry on the characteristics of the adsorbent surface, such as the charge and functional groups, has yet to be examined but deserves additional investigation in the future. Previous studies have indicated that the branched G units exhibit a higher affinity for various molecules, including cellulase and MO, than linear S units [7]. Consequently, the increased branched G lignin, rather than the linear S lignin, may contribute to the enhanced porosity and total adsorption volume of the porous carbon material prepared from BpF5H_KO plants, improving its adsorption capacity for MO. Additionally, the heterogeneity and complexity of lignin impact the selectivity and yield during the conversion of lignocellulosic biomass to value-added chemicals [8]. The increased G units resulting from *F5H* mutation in paper mulberry wood may negatively alter the selective scission of cross-linked C-C and C-O bonds in the BpF5H_KO lignins. Thus, the BpF5H_OE lignins with reduced G units and increased S units are still valuable materials for generating lignocellulosic biomass-based high-value chemicals with low cost.

In summary, employing the recently developed CRISPR/Cas9-mediated gene editing technology [9, 10] we generated a BpF5H mutant and altered the biosynthesis of S and G lignins in paper mulberry, leading to increased adsorption capacity of adsorbate molecules in their corresponding porous carbon. These findings underscore the potential of *F5H* as a promising target for developing high-value germplasm through lignin bioengineering not only in paper mulberry and but also in other wood species.
